# Impact of Combined Heat and Drought Stress on the Potential Growth Responses of the Desert Grass *Artemisia sieberi alba*: Relation to Biochemical and Molecular Adaptation

**DOI:** 10.3390/plants8100416

**Published:** 2019-10-15

**Authors:** Haifa Abdulaziz S. Alhaithloul

**Affiliations:** Biology Department, College of Science, Jouf University, Sakaka 2014, Saudi Arabia; haifasakat2030@gmail.com

**Keywords:** plant abiotic stress, agronomy, photosynthesis, WUE, gene expression, secondary metabolites, osmolytes, xerophytes

## Abstract

*Artemisia sieberi alba* is one of the important plants frequently encountered by the combined effect of drought and heat stress. In the present study, we investigated the individual and combined effect of drought and heat stress on growth, photosynthesis, oxidative damage, and gene expression in *A. sieberi alba*. Drought and heat stress triggered oxidative damage by increasing the accumulation of hydrogen peroxide, and therefore electrolyte leakage. The accumulation of secondary metabolites, such as phenol and flavonoids, and proline, mannitol, inositol, and sorbitol, was increased due to drought and heat stress exposure. Photosynthetic attributes including chlorophyll synthesis, stomatal conductance, transpiration rate, photosynthetic efficiency, and chlorophyll fluorescence parameters were drastically reduced due to drought and heat stress exposure. Relative water content declined significantly in stressed plants, which was evident by the reduced leaf water potential and the water use efficiency, therefore, affecting the overall growth performance. Relative expression of aquaporin (*AQP*), dehydrin (*DHN1*), late embryogenesis abundant (*LEA*), osmotin (*OSM-34*), and heat shock proteins (HSP70) were significantly higher in stressed plants. Drought triggered the expression of *AQP*, *DHN1*, *LEA*, and *OSM-34* more than heat, which improved the HSP70 transcript levels. *A. sieberi alba* responded to drought and heat stress by initiating key physio-biochemical and molecular responses, which were distinct in plants exposed to a combination of drought and heat stress.

## 1. Introduction

Several reports have stated that many areas of the world will suffer from drought in the coming decades due to climatic changes [1,2,3,4]. On one hand, there is an increasing demand for water, and on other hand, seasonal climatic fluctuations, and an apparent decline in available natural water and global increases in CO_2_ are all occurring [3,4]. These factors significantly threaten the floristic diversity coverage in the arid ecosystems, and these effects become more drastic due to the coexistence of heat and drought [5,6,7,8,9]. The combined effect of stresses alters different agronomic characteristics by influencing the biochemical and physiological functions, thereby influencing plant growth, development, and yield [2,4,9,10]. Drought and heat stress affect the phonological traits that have a pivotal role in the adaptation of plants to counteract adverse environmental factors [11,12,13]. To prevent stress-induced oxidative damage, plants improve their antioxidant defense system to scavenge reactive oxygen species (ROS) [14,15,16,17,18,19]. Photosynthetic machinery, quantum yield efficiency (Fv/Fm), and activity of PSII decrease significantly after either heat or drought stress exposure [20,21,22] or both combined [23]. High temperature and drought inhibit seed filling and seed production [24,25,26], and gene expression changes in floral organs [19,27,28]. Combined exposures to temperature and drought stress limit the growth of spring wheat by limiting the leaf chlorophyll content, grain number, and harvest index [29,30,31]. Moreover, heat and drought stress cause down-regulation of respiration and metabolites correlated to the tricarboxylic acid cycle pathways in corn [32]. Therefore, it is inferred that, in the future, temperature and drought stress will cause a significant decline in food production, posing a threat to world food security [33].

Osmoregulation and ion homeostasis through compartmentalization are adaptive mechanisms contributing enormously to stress tolerance [34,35]. Under stressful conditions plant cells synthesize and accumulate higher concentrations of compatible metabolites, such as soluble sugars, and certain amino acids, such as proline and glycine betaine (GB), leading to a reduction of the osmotic potential [36,37,38]. Elevated levels of osmolyte accumulation in plant cells have been correlated with enhanced stress tolerance through the scavenging of free radicals and enzyme protection [29]. Furthermore, secondary metabolite production also undergoes dramatic change and plays an essential role in many complex biotic and abiotic interactions [24,25,26]. Recently, the plant secondary metabolites received significant attention and many reports suggest its pivotal role in plant stress physiology [24,25,26]. Molecular and gene expression changes in plants exposed to combined stresses have been observed in different plants, endorsing a number of antioxidant enzymes as a defense mechanism to ameliorate them from the injurious effect induced by free radicals [19,26,27]. Therefore, further studies are needed to provide a better and a deep understanding of protection mechanisms within combined stress that in turn will improve the yield.

*Artemisia sieberi alba* belongs to Asteraceae and is commonly known as a desert or white wormwood. It is used in folk medicine as an antiseptic, antispasmodic, and vermifuge [39]. *A. sieberi alba* contains a high percentage of phytochemical compounds such as herbalbin, cis-chryanthenyl acetate, flavonoids including hispidulin and cirsilineol, and several terpenes like monoterpenes and sesquiterpenes [39]. In the last few years, heat waves and lower precipitation have led to a significant decline in the growth of *A. sieberi alba* and altered its physio-biochemical and molecular profile. Therefore, the present study was carried out to investigate (i) the combined effect of temperature and water stress on growth and photosynthesis of *A. sieberi alba* and (ii) physiological–biochemical and molecular traits that will be triggered to improve the growth performance under these stressful conditions.

## 2. Results

The effects of abiotic stresses, i.e., drought (D) and heat (H) alone and combined (D + H) on *A. sieberi alba* plants were analyzed for various morphological, growth, physiological, biochemical, phytochemical, and gene expression parameters.

**Figure 1:**
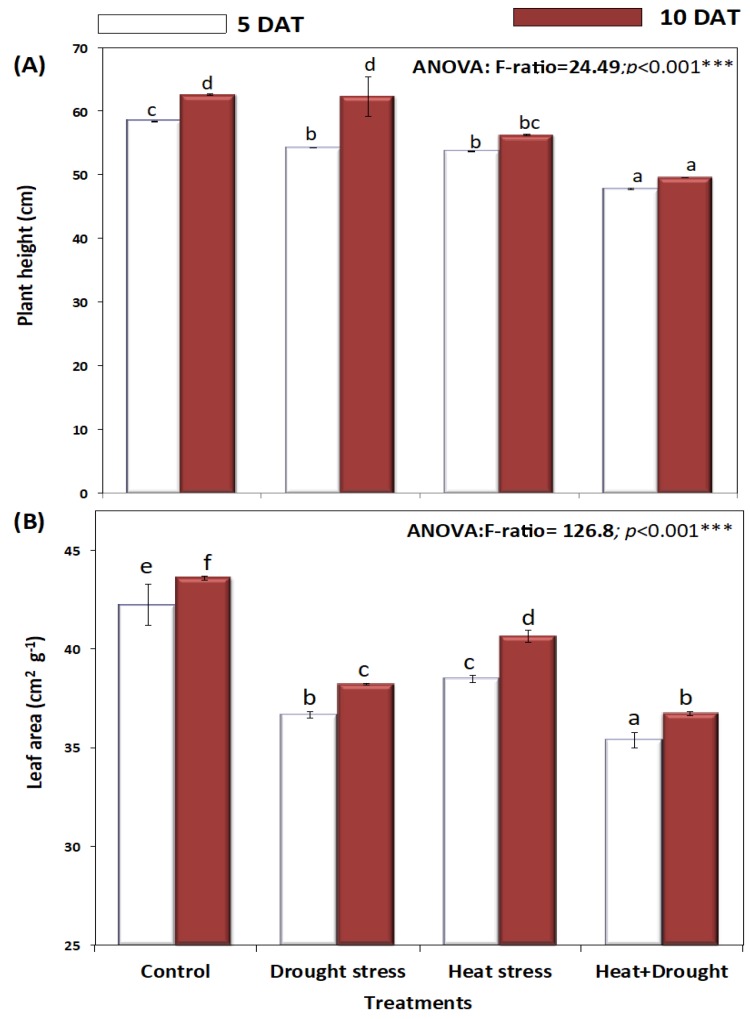
Effect of drought, heat, and interaction between drought and heat stress on (**A**) plant height (cm) and (**B**) leaf area (cm^2^ g^−1^) of *A. sieberi alba.* Data expressed as mean of triplicates, error bars represent standard error for means. Means marked with different letters are significantly different according to ANOVA and Duncan’s multiple range comparisons (DMRTs) at *p* < 0.05.

**Figure 2:**
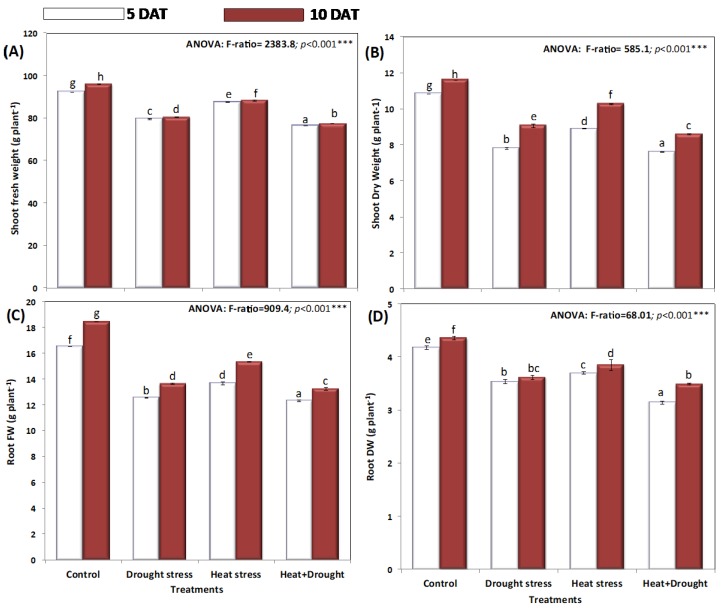
Effect of drought, heat, and interaction between drought and heat stresses on (**A**) shoot fresh weight (g-FW plant^−1^), (**B**) shoot dry weight (g-DW plant^−1^), (**C**) root fresh weight (g-FW plant^−1^), and (**D**) root dry weight (g-DW plant^−1^) of *A. sieberi alba.* Data expressed as mean of triplicates, error bars represent standard error for means. Means marked with different letters are significantly different according to ANOVA and DMRTs at *p* < 0.05.

**Figure 3:**
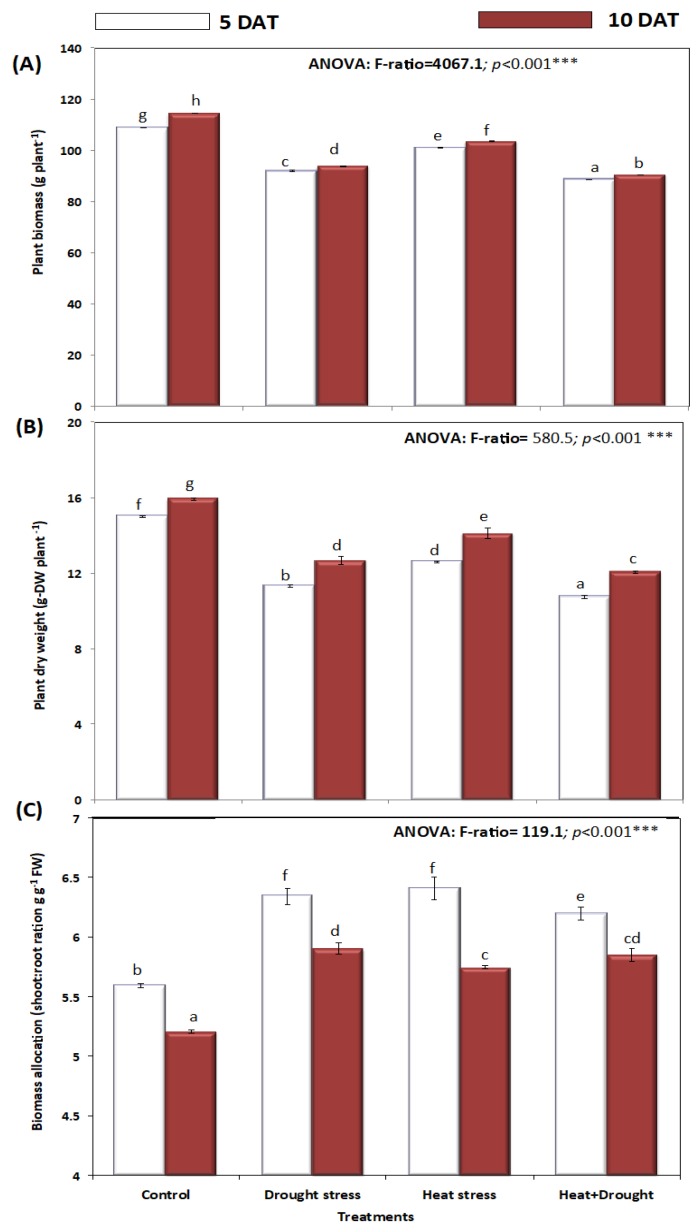
Effect of drought, heat, and interaction between drought and heat stresses on (**A**) plant biomass (g-FW plant^−1^), (**B**) plant dry weight (g DW plant^−1^), (**C**) biomass allocation (shoot-root ratio, g g^−1^ FW) of *A. sieberi alba.* Data expressed as mean of triplicates, error bars represent standard error for means. Means marked with different letters are significantly different according to ANOVA and DMRTs at *p* < 0.05.

Morphological and growth parameters of *A. sieberi alba*, such as plant height, leaf area, shoot and root fresh and dry weight, plant biomass, and biomass allocation, under D, H, and D + H conditions, are presented in Figure 1, Figure 2 and Figure 3. *A. sieberi alba* grown under a control condition maintained proper plant height which were 58 and 62 cm at five and 10 days, respectively (Figure 1A). However, plants exposed to high temperatures (37 °C) for five and ten days exhibited a significant decline in plant height as compared with the untreated control. Furthermore, drought stress in *A. sieberi alba* plants, induced by restricting watering, showed heights of 54 and 62 cm, and the simultaneous effect of drought and heat stress decreased plant height to 47 and 50 cm after five and ten days of stress treatments, respectively (Figure 1A).

Leaf area is another essential parameter used to monitor plant growth under environmental stresses, including heat and drought. Leaf area data are depicted in Figure 1B, and the difference between treatment groups, including the control, was assessed by using ANOVA and Duncan’s multiple range comparisons (DMRTs). Heat and drought alone significantly decreased *A. sieberi alba* leaf area, however, combined stress (H + D) severely decreased the leaf area of *A. sieberi alba* (Figure 1B).

The shoot fresh weight of *A. sieberi alba* plants grown under normal environmental conditions (17–22 °C) significantly increased from 92.3 g plant^−1^ to 95.9 g plant^−1^ after ten days. However, exposure of *A. sieberi alba* plants to abiotic stress (drought and heat stresses) decreased the shoot fresh weight (SFW) to 79.4, 87.5, and 76.2 g plant^−1^ in plants stressed with drought, heat, and drought and heat stresses, respectively, for five days, and the same trend was observed after ten days (Figure 2A). Ten days after stress, the SFW of *A. sieberi alba* plants decreased significantly to 80.3, 88.2, and 77.3 g plant^−1^ for drought, heat, and drought and heat stresses, respectively. Accordingly, the shoot dry weight after ten days of stress exposure declined from 11.6 g plant^−1^ (control) to 9.07, 10.2, and 8.6 g plant^−1^ in plants stressed with drought, heat, and drought and heat stress, respectively, for ten days (Figure 2B).

Plant growth with both abiotic stresses recorded the lowest minimum values of the shoot and root biomass, consequently affecting the whole plant biomass (Figure 3A,B). Plants generally allocate biomass according to their needs, however, under stresses, *A. sieberi alba* plants showed a significant modification in their biomass allocation in terms of shoot and root ratios. Generally, biomass allocation increased with the abiotic stresses, especially temperature, drought, and temperature and drought stresses. A marked decrease was observed in root biomass relative to the decrease in shoot biomass, reflected in increased shoot-root (S:R) ratios (Figure 3C). The highest recorded biomass allocation was 5.9 (S:R ratio, g g^−1^ FW). Differences between treatments in biomass allocation in *A. sieberi alba* plants was assessed by two-way analysis of variance followed by Duncan’s multiple range comparisons (DMRTs).

**Figure 4:**
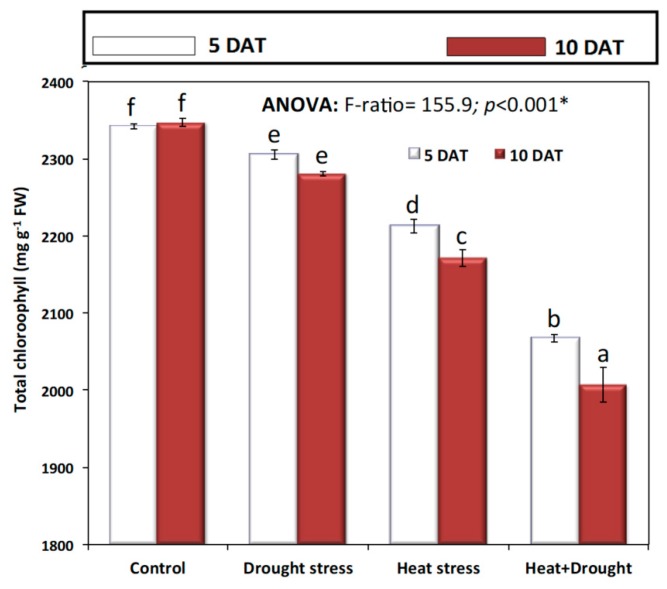
Effect of drought, heat, and interaction between drought and heat stresses on total chlorophyll contents of *A. sieberi alba*. Data expressed as mean of triplicates, error bars represent standard error for means. Means marked with different letters are significantly different according to ANOVA and DMRTs at *p* < 0.05.

**Figure 5:**
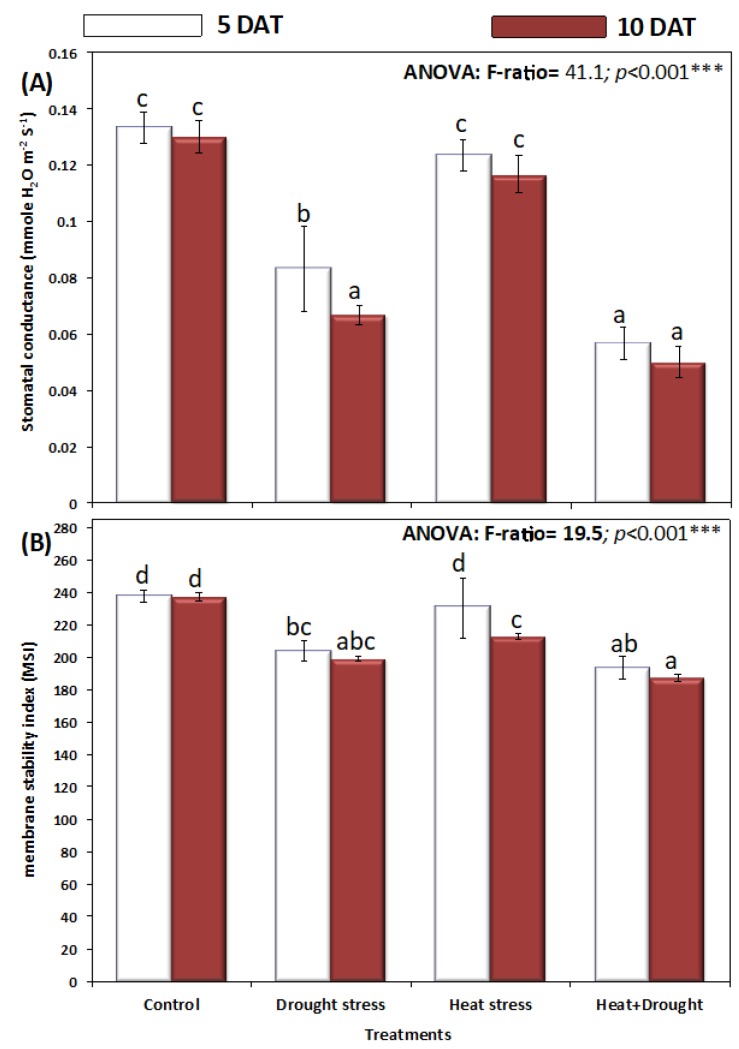
(**A**,**B**) Effect of drought, heat, and interaction between drought and heat stresses on (**A**) stomatal conductance and (**B**) membrane stability index (MSI) of *A. sieberi alba.* Data expressed as mean of triplicates, error bars represent standard error for means. Means marked with different letters are significantly different according to ANOVA and DMRTs at *p* < 0.05.

*A. sieberi alba* plants exposed to drought and temperature stress individually, as well as combined, showed a significant decline in chlorophyll content. The reduction was more obvious as the period of stress increased from five to ten days (Figure 4). Individually, heat stress resulted in a more significant (*p* ≤ 0.05) decline in total chlorophyll content as compared with drought stress, and the decline reached a maximum in plants exposed to drought and heat stress (Figure 4). Stomatal conductance (mmole H_2_O m^−2^ S^−1^) was significantly decreased by drought and temperature stress at both five and ten days after treatment (Figure 5A).

Stomatal conductance in the leaves of the untreated control *A. sieberi alba* plant was 0.13 mmole H_2_O m^−2^ S^−1^, although it significantly decreased to 0.08 and 0.06 in plants stressed with drought stress and both heat and drought. Drought stress proved more damaging in reducing stomatal conductance, however, heat stress caused a nonsignificant change in stomatal conductance of *A. sieberi alba*, which was 0.12 mmole H_2_O m^−2^ S^−1^ (Figure 5A). Membrane stability index (MSI) is an important measure of plant membrane behavior under heat and drought stresses, and the results of the membrane stability index are shown in Figure 5B. The MSI of *A. sieberi alba* plants significantly (*p* < 0.05) decreased under drought and drought and heat stresses, however, was not altered significantly (*p* > 0.05) under heat stress alone (Figure 5B).

**Figure 6:**
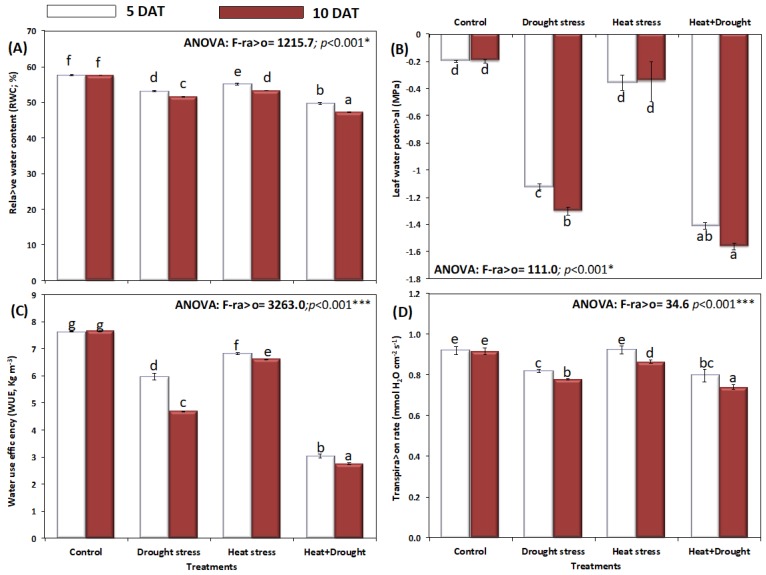
Effect of drought, heat, and interaction between drought and heat stresses on (**A**) relative water contents (RWC %), (**B**) leaf water potential (MPa), (**C**) water use efficiency (WUE), and (**D**) transpiration rate of *A. sieberi alba.* Data expressed as mean of triplicates, error bars represent standard error for means. Means marked with different letters are significantly different according to ANOVA and DMRTs at *p* < 0.05.

The results depicting the effect of drought and temperature stress on plant water status, evaluated in terms of Ψ_pd_, RWC, and WUE are shown in Figure 6A–D. Stress exposure induced a significant decline on relative water content (RWC), leaf water potential (MPa), water use efficiency (WUE, kg m^−3^), and transpiration rate (mmole H_2_O cm^−2^ S^−1^), with a more apparent decline after ten days of stress. Individually the decline was more in drought-stressed plants as compared with temperature-stressed plants. This decline in relative water content (RWC), water use efficiency (WUE, kg m^−3^), and transpiration rate (mmole H_2_O cm^−2^ S^−1^) was significantly less after exposure to both (drought and heat) abiotic stresses (Figure 6A,C,D). Consequently, the leaf water potential decreased markedly (*p* < 0.05) in plants exposed to drought and combined stress (D + H).

**Figure 7:**
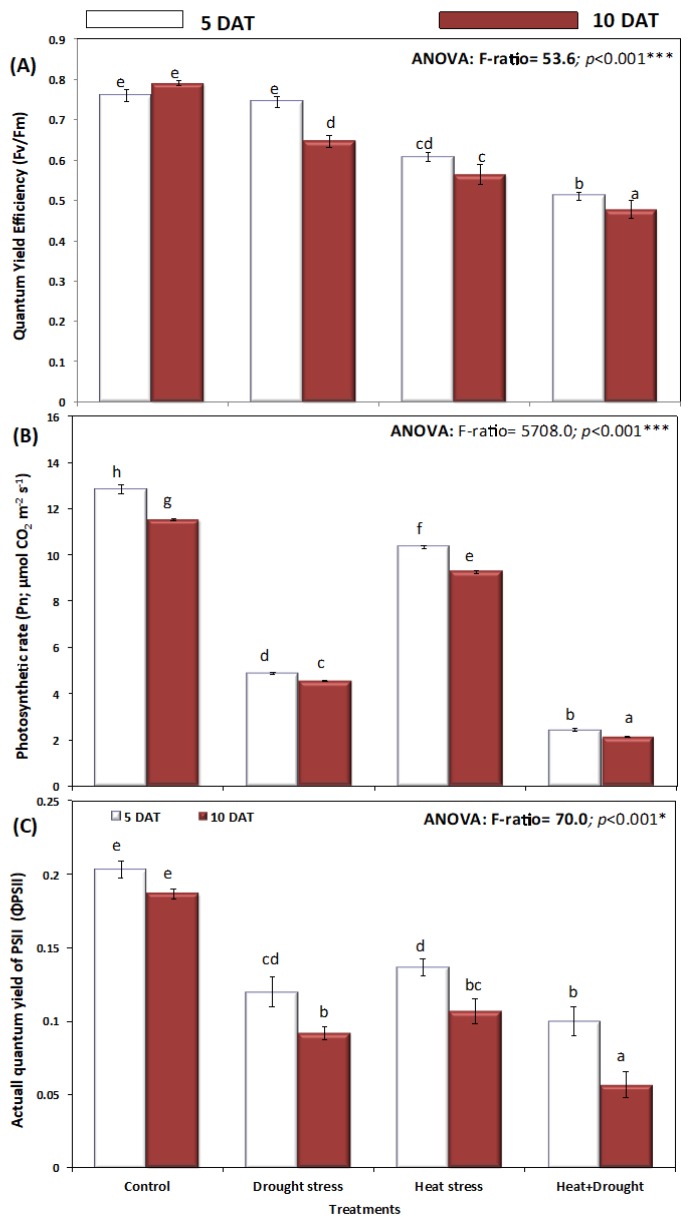
Effect of drought, heat, and interaction between drought and heat stresses on (**A**) quantum yield efficiency (*Fv/Fm*), (**B**) photosynthetic rate (Pn), and (**C**) actual quantum yield efficiency (ΦPSII) of *A. sieberi alba*. Data expressed as mean of triplicates, error bars represent standard error for means. Means marked with different letters are significantly different according to ANOVA and DMRTs at *p* < 0.05.

**Figure 8:**
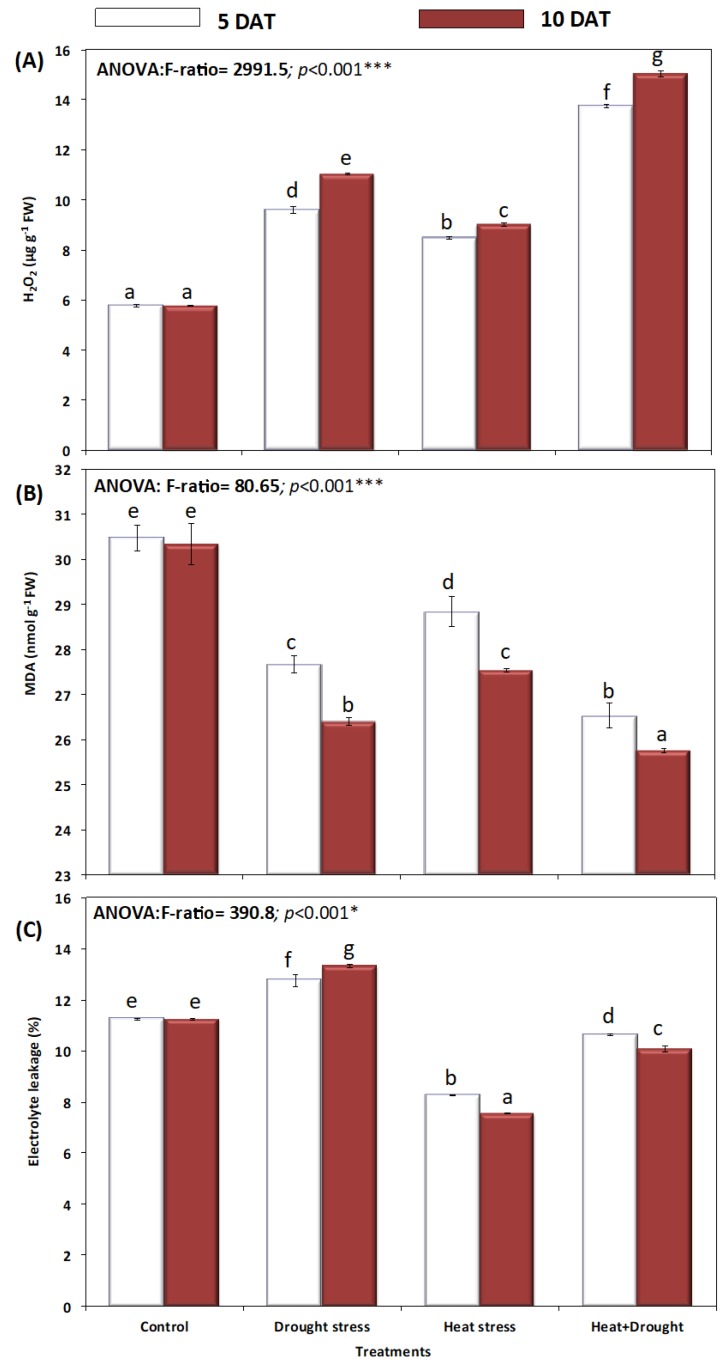
Effect of drought, heat, and interaction between drought and heat stresses on oxidative damage (**A**) cellular hydrogen peroxide level (H_2_O_2_), (**B**) lipid peroxidation (MDA), and (**C**) electrolyte leakage (%) (EL) of *A. sieberi alba*. Data expressed as mean of triplicates, error bars represent standard error for means. Means marked with different letters are significantly different according to ANOVA and DMRTs at *p* < 0.05.

Drought, heat, and the combination of both decreased the photosynthetic quantum yield efficiency (Fv/Fm), the actual quantum yield of PSII, and the photosynthetic rate (Pn) (Figure 7). The decline in quantum yields in *A. sieberi alba* plants under stress (H, D, and H + D) increased with time, from five days to ten days of stress treatment. The lowest quantum yields were recorded in plants under the combined effect of H + D for five and ten days, respectively (Figure 7A). Moreover, untreated (unstressed) plants maintained high leaf photochemical efficiency, Fv/Fm, chlorophyll content, photosynthetic rate, and PSII quantum yield. Drought, heat, and drought and heat significantly (*p* ≤ 0.05) reduced photosynthetic rate (Pn) and actual quantum yield of PSII (Figure 7B,C). Individually, drought stress decreased the photosynthetic rate and actual quantum yield of PSII more than heat stress. The maximal decline in photosynthetic rate and actual quantum yield of PSII (ΦPSII) was observed in H + D stressed plants (Figure 8B,C).

Hydrogen peroxide accumulation was significantly induced by drought and heat stresses, resulting in higher H_2_O_2_ accumulation in plants exposed to combined stress (Figure 8A). The drought and heat stress-induced enhancement in H_2_O_2_ resulted in a significant increase in leaf electrolyte leakage (EL) and lipid peroxidation (MDA accumulation), and also in a reduced membrane stability index (MSI, described above). MDA decreased significantly (*p* ≤ 0.05) in plants exposed to heat, drought, and combined stress (Figure 8B). Combined stress significantly induced a substantial decrease in MDA accumulation and electrolyte leakage revealed a significant (*p* ≤ 0.05) increase during drought, however, EL decreased significantly (*p* ≤ 0.05) in *A. sieberi alba* plants under heat and combined H + D stresses (Figure 8C).

**Figure 9:**
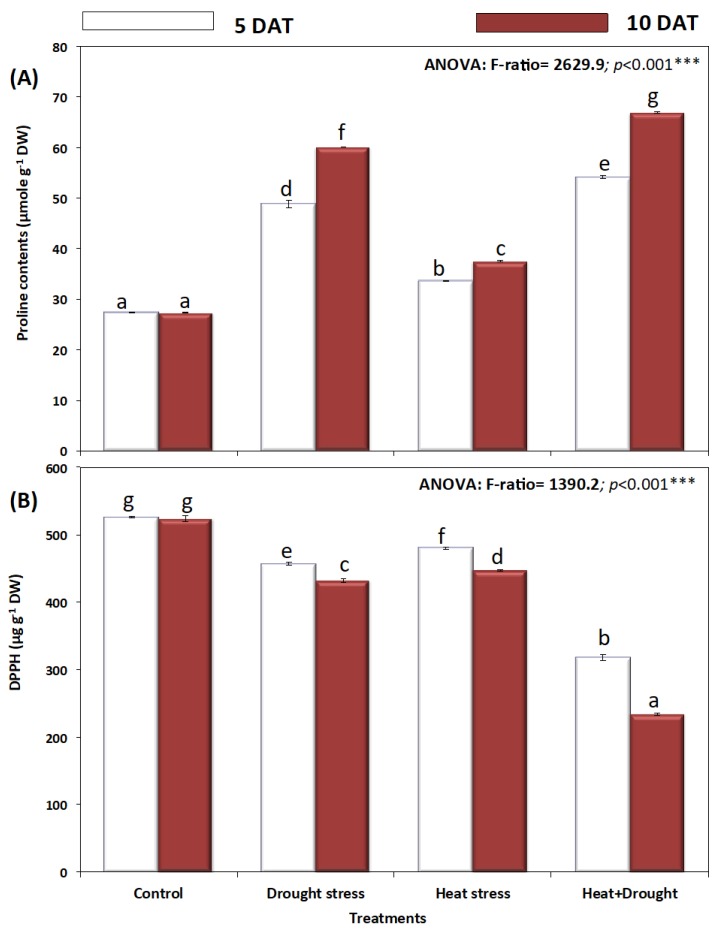
Effect of drought, heat, and interaction between drought and heat stresses on (**A**) proline content and (**B**) Total antioxidant estimated by DPPH free radical scavenging activity of *A. sieberi alba.* Data expressed as mean of triplicates, error bars represent standard error for means. Means marked with different letters are significantly different according to ANOVA and DMRTs at *p* < 0.05.

**Figure 10:**
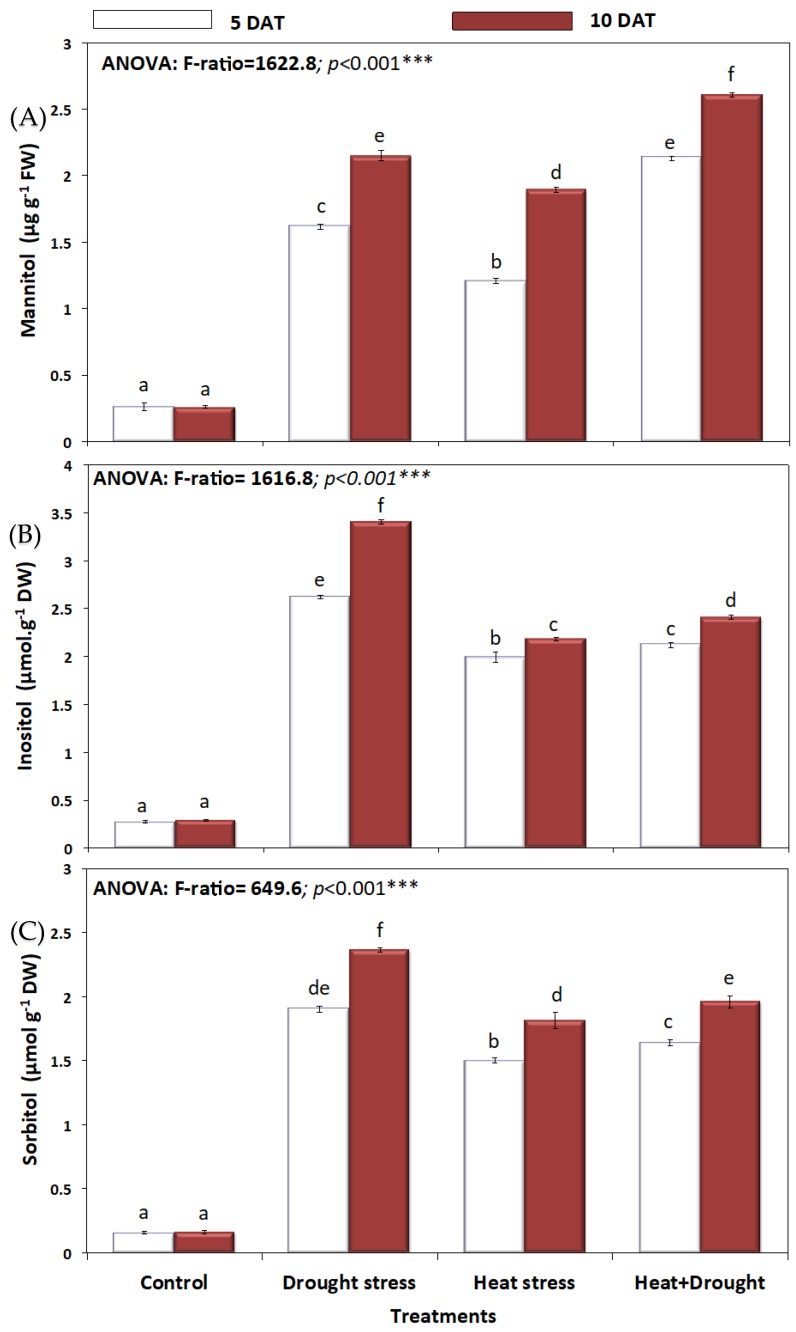
Effect of drought, heat, and interaction between drought and heat stresses on (**A**) mannitol (µg g^−1^FW), (**B**) inositol (µmol. g^−1^FW), and (**C**) sorbitol (µmol. g^−1^FW) of *A. sieberi alba.* Data expressed as mean of triplicates, error bars represent standard error for means. Means marked with different letters are significantly different according to ANOVA and DMRTs at *p* < 0.05.

*A. sieberi alba* plants exposed to drought and heat stress accumulated significantly more compatible osmolytes than control plants (Figure 9A and Figure 10). All osmotic solutes including proline, mannitol, inositol, and sorbitol were higher in drought-stressed plants, followed by their heat-stressed counterparts. In response to drought, heat and combined (H + D) stress, the leaf proline content was significantly (*p* ≤ 0.05) increased at either five days or ten days (Figure 9A). The highest increase in leaf proline was observed in drought and drought and heat-stressed plants. Leaf polyols (mannitol, inositol, and sorbitol) in *A. sieberi alba* plants significantly (*p* ≤ 0.05) increased under all stress conditions (H, D, and H + D) (Figure 10A–C). The highest increase in mannitol content was observed in the combined stressed group, which was significantly (*p* ≤ 0.05) increased by seven-fold over the untreated control (Figure 10A). Drought stress induced the highest increase (*p* ≤ 0.05) in leaf inositol and sorbitol content, which continued from five to ten days of stress (Figure 9B,C).

The DPPH radical scavenging activity of different treatments in *A. sieberi alba* leaves revealed a decrease (Figure 10B). The combined effect of heat and drought induced a decline of 40% in DPPH radical scavenging activity, with a more pronounced effect after ten days of stress treatments (Figure 9B).

*A. sieberi alba* as an important wild medicinal plant with various valuable phytochemical constituents, therefore, an evaluation of the phytochemical composition of *A. sieberi alba* leaves was carried out to assess the effect of abiotic stress. The phytochemical constituents screened included flavonoids, tannins, phenols, saponins, glycoside, alkaloids, steroids, terpenoids, soluble sugars, and sterols. As a response to environmental stresses including drought, heat, and drought and heat, the phytochemical compositions of *A. sieberi alba* plants changed considerably and significantly (Table 1). Combined stress (H + D) increased various phytochemical compounds including flavonoids, tannins, phenols, saponins, glycoside, alkaloids, steroids, and terpenoid, however, there was no change from the untreated control in the content of soluble sugars and sterols. Heat stress significantly induced an increase in the content of tannins, alkaloids, terpenoids, and steroids, however, drought stress induced higher levels of saponins and steroids (Table 1).

**Figure 11:**
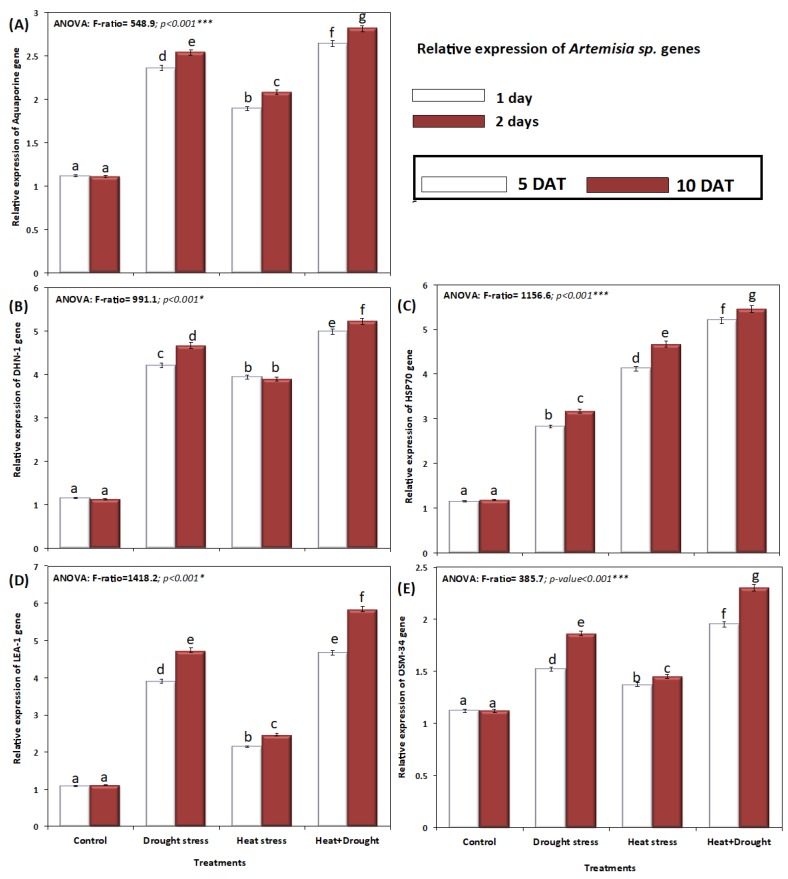
Relative gene expression of *A. sieberi alba.* Under the effect of drought, heat, and interaction between drought and heat stresses on (**A**) aquaporin gene, (**B**) DHN-1 gene, (**C**) HSP-70 gene, (**D**) LEA-1 gene, and (**E**) OSM-34 genes. Data expressed as mean of triplicates, error bars represent standard error for means. Means marked with different letters are significantly different according to ANOVA and DMRTs at *p* < 0.05.

The relative gene expression of aquaporins, *DNH1*, *HSP70*, *LEA1*, and *OSM-34* genes was performed in *A. sieberi alba* under drought and heat stress conditions by real-time (RT)-qPCR (Figure 11A–E). Expression of the aquaporin gene (*AQP*) was performed by using the relative quantification of *SsPIP1 aquaporin-1* (*unit SD264077*, Table 1). Drought stress and combined stress significantly induced *AQP* relative gene expression as revealed by the analysis of variance (Figure 11A). Generally, the relative expression of *AQP*, *DNH1*, *HSP70*, *LEA1*, and OSM-*34* was significantly increased under both stress conditions, attaining maximal values in plants exposed to drought + heat stress (Figure 11). Individually, expression of *AQPs*, *DNH1*, *LEA1*, and *OSM-34* was higher in drought-stressed plants than HSP70, which showed higher expression levels in heat-stressed plants. Dehydrin (*DHN1* and *LEA1*) genes exhibited a significant increase under drought and drought and heat stress conditions, where a cellular protective role during stress was encoded by *DHNs* and *LEA* (Figure 11B,D). With an increase in the stress period from five to ten days, the relative expression of all genes showed a gradual increase (Figure 11).

## 3. Discussion

Drought and heat stress were considerably more anxious for plants as compared with either stress alone, indicating that environmentally associated heat waves which are generally associated with arid durations in summer and spring might be deleterious to *A. sieberi alba* [39]. To neutralize these negative impacts, plants initiated several key mechanisms, with common reactions to individual or combined stresses [40]. In this study we investigated responses at the physiological, biochemical, and molecular levels to drought and temperature stress focusing on the combined effect of drought and temperature stress. High temperature accelerates the depletion of soil water, probably by increased evaporation and transpiration, which was evident in this study. Furthermore, it was observed that both stresses imparted serious phenotypical modifications. Heat treatment exerted more significant impact than drought on physiological parameters such as height, chlorophyll content, and photosynthesis reflected in the differences in responses and stress pathways. It has been reported that growth and functioning of shoot and root are reduced resulting in considerable changes in the distribution of essential components from root to shoot [41]. It has been shown that several plant hormones and nutrient availability regulate key physiological pathways under stresses [42,43,44]. The phenology relationship and water use are main indicators for drought stress [45]. We found that *A. sieberi alba* exhibited significant decline in the morphological parameters and the water use efficiency, with the effect being much more apparent in plants exposed to combined drought and heat stress.

In this study, dry matter declined due to temperature and drought stress. Yield and biomass accumulation in plants depend on the number of plants, the production of dry matter, as well as the number and size of seeds. Earlier, it has been reported that high temperatures and water stress decline the yield by affecting the growth through reduced light interception over the shortened life cycle [5]. Dreesen and his co-author [46] have also confirmed that, the combined effect of high temperatures and water stress on crop growth and yields is more damaging than the individual stress. Similar to our results, drought and temperature effects have been reported to reflect reduced plant biomass accumulation, shorter internodes, early senescence and death, and fruit discoloration [44,47]. Growth is severely affected due to alterations in the physiological and metabolic pathways, for instance photosynthesis and related attributes including chlorophyll production and fluorescence, stomatal behavior, sugar synthesis, and metabolism, in addition to the water relations restricting the allocation of sucrose to developing seeds thereby affecting their size and number [10,48]. In addition, understanding the influence of drought and high temperature on the functioning of related enzymes and hormones may be helpful to unraveling the exact mechanisms involved.

The combined effect of drought and heat stress on *A. sieberi alba* significantly affected RWC and leaf water potential. Reduced RWC affects the cellular functioning adversely and our results are in corroboration with the finding of [49] for barley and [48] for chickpea. Extensive damage to membranes in terms of electrolyte leakage, reduced chlorophyll content, and photosynthetic performance after exposed to combined stresses is attributed to the substantial reduction of leaf RWC and the stomatal conductivity [48]. Drought and temperature stress increased the electrolyte leakage level, indicating membrane instability which could be due to alterations in the lipid–protein configuration [50] and loss of cellular functioning [51]. In accordance to our results, earlier studies which discussed the deleterious impact of combined stress on RWC and membrane leakage in chickpeas [52] and *Poa pratensis* L. are available [53,54,55,56]. The damaging impact of drought and high temperature are obvious on the functioning of photosystem II (Fv/Fm) and the maximum quantum yield [57]. Stress induced reduction in the electrolyte leakage indicates the degradation of the D1 protein configuration [58,59] and loss of cellular functioning [47,60,61].

Negative effects of drought and heat stress may have considerably contributed to reduce photosynthetic functioning reflecting on the altered physiological process and plant metabolism [62]. Further drought and heat stress declined chlorophyll concentration probably due to disturbances in chloroplast structural integrity, uptake of magnesium [57], and increased chlorophyll denaturation [63,64,65,66]. Increased stomatal conductivity during heat stress is considered to be an adaptive mechanism to improve transpiration for allowing cooling, as have been reported in wheat [67]. In this study, stomatal conductance in heat stressed plants was comparable with the controls, reflecting the reduction in stomatal conductance maximally due to the drought stress mediated declined leaf water potential [68,69] and the decline is highly intensified after the stress period prolonged from five to ten days.

Improved accumulation of secondary metabolites may have further strengthened the antioxidant potential leading to protection of the structural and functional integrity of thylakoid membranes and chlorophyll stabilization [70,71]. In this study, drought induced the accumulation of phenols more conspicuously than heat stress. Improved antioxidant potential reduces the oxidative damage to membranes and proteins hence protecting the organelle functioning and the whole plant performance [72,73]. Moreover, the interactive effects of heat and drought can be attributed to the improved scavenging capacity of antioxidants triggered by both stress factors. It has been reported that stress induced oxidative injury results from the disequilibrium between generation and elimination of free radicals in the photosynthetic and respiratory pathways [74,75,76]. It is believed that increased metabolite (phenolics, flavonoids, anthocyanins, lignin, etc.) synthesis strengthens the non-enzymatic antioxidant system [44,47,77,78]. Additionally, to the enzymatic components, the non-enzymatic components including phenols, flavanols, ascorbic acid, glutathione, and tocopherols, also contribute to prevention of oxidative stress effects through improved ROS scavenging [79]. Sayed et al. [80] found that flavonoids impart photoprotection in plants against high temperatures and drought stress. Reports discussing the combined effect of drought and heat stress on the accumulation of phenols and antioxidant potential are rare. Improved antioxidant potential reduces the oxidative damage to membranes and proteins, hence protecting the organelle functioning and the whole plant performance [72,73]. The interactive effects of heat and drought can be attributed to the improved scavenging capacity of antioxidants triggered by both stress factors [78,79]. 

Secondary metabolites significantly affect the plant interactions with biotic and abiotic components in addition to their key role in medical, nutritional, and cosmetic purpose [26,27]. To ease stress mediated deleterious effects plants increase the synthesis of phenolics, flavonoids, alkaloids, terpenoids, steroids, tannins, saponins, glycosides, and xanthoprotein [44,47]. It is believed that increased metabolite synthesis strengthens the non-enzymatic antioxidant system by altering peroxidation kinetics and maintaining the membrane fluidity [77,78]. Additionally, phenols, xanthoprotein, and flavonoids impart photoprotection in plants against damaging growth factors like radiations [81]. The accumulation of secondary metabolites may have reduced the oxidative damage effects by limiting the accumulation of ROS under both individual as well as combined stresses [80,81,82,83,84,85]. Under water and UV stressed conditions, flavonoid synthesis and ROS scavenging has been reported to increase [86]. Similar to our observation’s, higher flavonoid, saponins, and tannins accumulation has also been reported in wheat exposed to drought and high temperature stress [79].

Furthermore, combined effect of drought and high temperature triggered the plants to accumulate significant quantities of low molecular weight compounds such as proline, glycine betaine, and sugar alcohols to buffer the cellular redox potential for better withstanding the stress factor through maintenance of tissue water content [36,82,87]. Accumulated sugars in stressed plants can serve cellular functions such as energy source for stress recovery, signal transduction, and osmoprotection [83]. Heat or water stressed plants accumulate soluble sugars to an appreciably high level in order to generate significant osmotic potential [84]. Proline acts as a metabolic signal leading to control the mitochondrial and photosynthetic functions by maintaining the redox balance, hence imparting stress tolerance and plant development [82,85]. Future studies to unravel the exact regulation at genetic and molecular levels are required.

A plant’s response to the combination of stress factors like drought and heat imparts suppression of key processes like photosynthesis with concomitant enhancement in the expression of defense protein coding genes [36,54]. Among the different genes worked out, the expression of aquaporin (AQP), DHN-1, LEA, and OSM-34 was much more apparent in drought stress conditions, whereas HSP-70 expression was much more apparent in heat stressed ones. Multiple isoforms of AQPs exist in plasmalemma and tonoplast membranes maintaining the flow of water in and out of cells ultimately influencing the water transfer via leaves and roots [88,89]. In addition, AQPs have been identified to play key roles in regulation of root and leaf hydraulic conductance, thereby influencing processes including phloem loading, xylem water exit, stomatal movement, and gas exchange [88]. Recently, Wang et al. [89] have demonstrated increased drought stress tolerance in potato over-expressing plasma membrane AQP gene *StPIP1*. Late embryogenesis abundant (LEA) proteins are important hydrophilic proteins having a major role in abiotic stress tolerance in plants, especially in drought [90]. LEA proteins mediate plant protection by serving as antioxidant, hydration buffering, stabilizing membranes and proteins, metal ion binding, and DNA and RNA interactions [91,92]. Dehydrin (DHN) proteins fundamentally control growth under abiotic stresses and it has been reported that plants exhibiting higher expression of DHN show improved tolerance to drought [93]. In *A. sieberi alba* the drought responsive genes including AQP, DHN, LEA, and OSM showed apparent enhancement in their expression under drought conditions as compared with heat stressed and the control, however, HSP70 transcript levels were more in heat stressed plants as compared with drought and the control. Plants exposed to heat stress exhibit protein dysfunction through their improper folding of amino acid chains to non-native proteins leading to unfavorable interactions and protein aggregation [94]. In the present study, higher transcript levels of HSP70 depict the role of these molecular chaperone for maintaining the high-quality proteins in the cell and also assist in cellular signaling. Therefore, upregulation of stress specific genes assisted *A. sieberi alba* in withstanding the drought and heat stress, and further studies are required to unravel their exact involvement in improving tolerance to combined drought and heat stress.

## 4. Materials and Methods

### 4.1. Pot Experiments and Stress Treatments

Achenes of *A. sieberi alba* were manually detached, and good seeds were detected by compound microscope. After germination, seedlings were commonly grown for three months. After that, pots were divided into four groups, including (a) control (normal irrigation at 17–22 °C), (b) drought, (c) heat (high temperature) stressed, and (d) drought and heat stressed, and were analyzed at five and ten days after stress treatment. For the drought group, water application was reduced by 50%. For the high-temperature stress group, 37 °C point was found to be suitable based on preliminary experiments. The pots for *A. sieberi alba*. were arranged in a completely randomized block design with five replicates in a greenhouse maintained at 65% humidity and a 12/12 h light/dark regime.

### 4.2. Growth Measurements

Morphological traits of treated and untreated *A. sieberi alba* plants were measured. Plant height was measured with a manual scale. Three plants, including the root, were harvested and taken to the laboratory to measure plant height, leaf area, shoot and root fresh and dry weights. Dry weight was measured after drying in an oven at 60 °C for 72 h.

### 4.3. Physiological Traits

#### 4.3.1. Relative Water Content and Leaf Water Potential (LWP)

For measurement of leaf, the relative water content (RWC) method described by [95] was followed. The following formula was used for calculation:

RWC (%) = (FW − DW)/(TW − DW) × 100

where FW = fresh weight, DW = dry weight, and TW = turgid weight.

Leaf water potential was measured in the last fully expanded leaf of control and stressed plants by using leaf water potential system WP4C, Germany.

#### 4.3.2. Gas Exchange, Chlorophyll Fluorescence Parameters and Water Use Efficiency

All photosynthetic measurements were performed on intact leaves on clear sunny days. Net photosynthetic rate (Pn), stomatal conductance (Gs), transpiration rate (Tr), and intercellular CO_2_ concentration (Ci) of fully expanded leaves were measured between 09.00 and 11.00 a.m. using an infrared gas analyzer system (TPS-2, USA). The CO_2_ concentration in the chamber was 380 ± 10 mol^−1^, and a photosynthetic photon flux density of 800 mol m^−2^ s^−1^ at the leaf surface was provided by a LED red-blue light source (LI-COR 6400–02). The water use efficiency (WUE) was calculated as Pn/Tr. Chlorophyll fluorescence measurements were made on the fully expanded leaves with the fluorometer after adapting in darkness for 30 min. Determination of Fv/Fm was based on the method of [96]. Steady-state fluorescence (Fs) and maximum fluorescence (Fm) of light-adapted leaves were measured when fluorescence reached a steady-state level. The maximum quantum efficiency of PSII photochemistry in the dark (Fv/Fm) and actual photosynthetic efficiency (ΦPSII open) in light were determined [97].

#### 4.3.3. Measurement of Chlorophyll

For estimation of chlorophyll pigments, fresh leaf sample (100 mg) was extracted in acetone, and the absorbance of the supernatant was recorded at 622, 664, and 440 nm using spectrophotometer [98].

### 4.4. Oxidative Stress Parameters

#### 4.4.1. Membrane Stability Index (MSI)

Membrane stability index was determined following the method of [99]. The 100 mg leaf samples were cut into discs and kept in test tubes containing 10 mL of double distilled water in two sets. One set was kept at 40 °C for 30 min and another set at 100 °C in boiling water bath for 15 min and their respective electric conductivities C_1_ and C_2_ were recorded. The calculation was done by the following formula:

Membrane stability index = [1− (C_1_/C_2_)] × 100

#### 4.4.2. Lipid Peroxidation

Lipid peroxidation was measured as malondialdehyde (MDA) content using the thiobarbituric acid method according to [100]. Molar coefficient of 155 mmol L^−1^ cm^−1^ was used for calculation and expressed as nmol g^−1^ FW.

#### 4.4.3. Hydrogen Peroxide

Hydrogen peroxide levels were determined by macerating 100 mg fresh tissue in 0.1% trichloroacetic acid (2 mL, TCA). Homogenate was centrifuged at 12,000× *g* for 15 min and 0.5 mL supernatant was mixed with 0.5 mL 10 mM potassium phosphate buffer (pH 7.0) and 1 M potassium iodide (1 mL). Absorbance was determined at 390 nm [101].

#### 4.4.4. Electrolyte Leakage

Electrolyte leakage was estimated by immersing leaf discs in deionized water in a test tube, and the initial electrical conductivity (ECa) was measured. The tissue containing tubes were heated for 25 min at 50 °C and 100 °C for 10 min in a water bath to measure the respective electrical conductivities (ECb) and (ECc), respectively [102]. The following formula was used for calculation:

$$\mathrm{Electrolyte}\;\mathrm{Leakage}\;\left(\%\right)=\;\frac{{\mathrm{EC}}_{b}-{\;\mathrm{EC}}_{a}}{{\mathrm{EC}}_{c}}\;\times \;100.\;$$

### 4.5. Determination of Proline Content

Proline was extracted by homogenizing one gram leaf samples in 3% sulphosalicylic acid. After that, 2.0 mL supernatant was mixed with 2 mL acid ninhydrin and glacial acetic acid and mixtures were incubated in a water bath at 100 °C for one h. After cooling, proline was separated using toluene and absorbance was measured at 520 nm [103].

### 4.6. Total Antioxidant Activity

Total antioxidant activity was estimated by measuring DPPH free radical scavenging activity using the method described by [104]. Plant tissue was extracted in ethanol (Merck, Darmstadt, German) and centrifuged at 10,000× *g* for 10 min. To 0.5 mL of supernatant was added 3 mL ethanol and 0.5 mM DPPH (300 μL) radical solution (Cayman Chemical Company, Michigan, USA) Therefore, absorbance was recorded at 517 nm. Ethanol and sample served as blank and control was ethanol and DPPH.

### 4.7. Determination of Mannitol, Sorbitol Inositol Content

The sorbitol content in treated and untreated *A. sieberi* plant leaves were measured using a Colorimetric Assay Kit (BioVision, Inc., San Francisco, CA, USA. Sorbitol was oxidized to fructose with the proportional development of intense color with an absorbance maximum at 560 nm. The sorbitol content in treated and untreated *A. sieberi* plant leaves were measured using a Mannitol Colorimetric Assay kit, (Sigma-Aldrich, Darmstadt Germany), measure the absorbance at 450 nm. The inositol content in the control and treated *A. sieberi* plant leaves were measured using a myo-Inositol Assay kit (BioVision, Inc., San Francisco, CA, USA.

### 4.8. Phytochemical Screening

Alkaloids were detected following Mayer’s Test after 2 mL of Mayer’s reagent was added to extract and formation of dull white precipitate revealed the presence of alkaloids.

For the testing of the presence of terpenoids, Hirshhorn reaction was followed, and the occurrence of red to purple color upon heating with trichloroacetic acid determined the presence of terpenoids.

For testing steroids, a Liebermann Burchard test was followed, and 1 mL extract was mixed with 1 mL of glacial acetic acid and 1 mL of acetic anhydride followed by the addition of two drops of concentrated sulphuric acid. The solution turned bluish green indicating the presence of steroids.

For tannins, ferric chloride was added to the extract, and the formation of a dark blue or greenish black color showed the presence of tannins.

Saponins were detected by the formation of copious lather after thoroughly shaking 1 mL of the extract with 5 mL of distilled water.

Flavonoids were detected by following the Shinoda test with the formation of a red color after the addition of magnesium and a few drops of concentrated hydrochloric acid.

For detection of phenols, a ferric chloride test was carried out and the extract was mixed with a few drops of aqueous ferric chloride (10%) and the appearance of blue or green color indicated the presence of phenols.

For detection of glycosides, the substance was mixed with a small amount of anthrone followed by addition of one drop of concentrated sulphuric acid. After gentle warming over a water bath, formation of dark green color indicated the presence of glycosides.

A xanthoprotein test was followed for detection of aromatic amino acids. The extract was mixed with 1 mL of concentrated nitric acid, and the white precipitate was formed. The mixture was boiled and cooled followed by the addition of 20% sodium hydroxide, and the appearance of orange color indicated the presence of aromatic amino acids.

### 4.9. RNA Isolation and Reverse Transcription of RNA (RT-PCR)

Total mRNA was isolated from 250 mg fresh leaves of *Artemisia* plants using Total RNA extraction kit (Sigma-Aldrich) according to the manufacturer’s protocol. The purified RNA was quantitated spectrophotometrically and analyzed on 1% agarose gel (Thermo Fisher Scientific, Waltham, MA, USA). Reverse transcription reactions were performed using oligo dT primer. The 20 μL reaction mixture contained 2.5 μL 5× buffer, 2.5 μL MgCl_2_, primer (10 pml/μL), 2.5 μL 2.5 mM dNTPs (QIAGEN, Hilden, Germany), 4 μL from oligo (dT), 0.2 μL (5 Unit/μL) reverse transcriptase (Promega, Madison, WI, USA), and 2.5 μL RNA. RT-PCR amplification was performed in a thermal cycler PCR (QIAGEN, Germantown, MD, USA), programmed at 42 °C for one h and 72 °C for 20 min.

### 4.10. Gene Expression Levels

The primer sequences used in qRT-PCR for the following five genes: HSP70, aquaporin, osmotin-34, LEA-1, and DHN1 genes are listed in Table 2 and β-actin was used as a reference gene. A total reaction volume of 20 µL was used including 2 µL of template, 10 µL of SYBR Green Master Mix (Fermentas, Burlington, ON, Canada), 2 µL of reverse primer (Fermentas, Burlington, ON, Canada), 2 µL of forwarding primer (Fermentas, Burlington, ON, Canada), and sterile distilled water. PCR assays were performed using the following conditions: 95 °C for 15 min followed by 40 cycles of 95 °C for the 30 s and 60 °C for 30 s. The CT of each sample was used to calculate ΔCT values (target gene CT subtracted from β-actin gene CT). The relative gene expression was determined using the 2^-ΔΔCt^ method [105].

### 4.11. Statistical Analysis

Data are presented as mean ± SEM (standard error for the mean) of three independent biological replicates. Statistical procedures were performed using IBM-SPSS version 23.0 for Mac OS and figures were compiled with Microsoft Excel 2016. Data were checked for outliers using SPSS. Data were checked for normality using Shapiro–Wilk’s normality test at *p* < 0.05 to assess whether the data were parametric or nonparametric. One-way and two-way analysis of variance (ANOVA), followed by Duncan’s Multiple Range Test (DMRTs) post hoc, were applied for each treatment group to estimate the significances among treatment groups. Means followed by different letters indicate significant differences at *p* < 0.05. Differences in the nonparametric data of phytochemical screening were assessed by using the Kruskal–Wallis significance test followed by pairwise comparisons post hoc analysis using SPSS. In order to integrate the results, a complete data set comprising all growth, physiological, biochemical, phytochemical, and gene expression parameters were subjected to multivariate analysis using SPSS statistical software.

## 5. Conclusions

Conclusively it can be inferred from the present study that drought and heat stress drastically influenced growth and metabolism of *A. sieberi alba* by reducing water uptake and use efficiency. Drought and heat stress inhibited photosynthesis and related attributes. Accumulation of osmolytes increased in stressed plants resulting in assisting in mitigation of oxidative effects of drought and heat stress on membrane structure and functioning. Differential expression of key drought and heat stress responsive genes was evident reflecting in some sort of dual functioning under combined effect of drought and heat stress, however, the interactive role of tolerance mechanisms at the biochemical and molecular levels in response to drought and heat stress is not known. Therefore, further studies are required to know the mechanisms involved in regulation of *A. sieberi alba* growth under the combined effect of drought and heat stress.

## Figures and Tables

**Table 1:** Effect of drought, heat, and combined effect of drought and heat stresses on estimated of phytochemicals in *A. sieberi alba*. leaves.

Treatments	Control	Drought (D)	Heat (H)	H+D	Kruskal-Wallis Test
Flavonoids	-	+	+	++ ^a^	<0.05*
Tannins	-	-	+ ^a^	+ ^a^	<0.05*
Phenols	+	+	+	++ ^a^	<0.05*
Saponins	-	+ ^a^	-	+ ^a^	<0.05*
Glycoside	+	+	+	++ ^a^	<0.05*
Alkaloids	+	+	++ ^a^	++ ^a^	<0.05*
Steroids	+	++ ^a^	++ ^a^	++ ^a^	<0.05*
Terpenoids	+	+	++ ^a^	++ ^a^	<0.05*
Soluble Sugars	-	-	-	-	*n.s.*
Sterols	-	-	-	-	*n.s.*

* significant at *p* < 0.05; n.s. nonsignificant difference at *p* > 0.05; ^a^ significantly different versus control group revealed by pairwise comparisons using the Kruskal–Wallis test.

**Table 2:** The sequences of the primers used in qRT-PCR.

Gene	Primer	Primer Sequence	Τm (°C)
β-Actin	For Rev	5′-GGTTCACTTGAAGGGTGGTG-3′ 5′-TGAGGTGTACCTGTCCTCGTT-3′	60
Aquaporin1 (*SsPIP1-1*)	For Rev	5′-GTTCCTATCCTTGCCCCACT-3′ 5′-AGGCGTGATCCCTGTTGTAG-3′	60
HSP 70	For Rev	5′-CAGATGAGGCCGTGGCTTAT-3′ 5′-GGGAGTCACATCCAACAGCAA-3′	60
Osmotin-34	For Rev	5′-GAACGGAGGGTGTCACAAAATC-3′ 5′-CGTAGTGGGTCCACAAGTTCCT-3′	60
LEA-1	For Rev	5′-CAGCGAAGTTTGGATGGAATG-3′ 5′-ACCTGTCGCCAATCAGAAGAT-3′	60
DHN1	For Rev	5′-GAGGAGGAGGGAGATGACGAAGAC-3′ 5′-GAGGAGGAGGGAGATGACGAAGAC-3′	60
